# Hispolon Decreases Melanin Production and Induces Apoptosis in Melanoma Cells through the Downregulation of Tyrosinase and Microphthalmia-Associated Transcription Factor (MITF) Expressions and the Activation of Caspase-3, -8 and -9

**DOI:** 10.3390/ijms15011201

**Published:** 2014-01-17

**Authors:** Yi-Shyan Chen, Shu-Mei Lee, Chih-Chien Lin, Chia-Yi Liu

**Affiliations:** 1 Department of Cosmetic Science, Providence University, 200 Chung-Chi Road, Shalu, Taichung 43301, Taiwan; E-Mails: chchlin@pu.edu.tw (C.-C.L.); g1000203@pu.edu.tw (C.-Y.L.); 2 Department of Cosmetic Science and Management, Mackay Medicine, Nursing and Management College, 92 Shengjing Road, Beitou, Taipei 11260, Taiwan; E-Mail: s107@eip.mkc.edu.tw

**Keywords:** apoptosis, caspase, hispolon, melanin, microphthalmia-associated transcription factor (MITF), tyrosinase

## Abstract

Hispolon is one of the most important functional compounds that forms *Phellinus linteus* (Berkeley & Curtis) Teng. Hispolon has antioxidant, anti-inflammatory, antiproliferative and anticancer effects. In this study, we analyzed the functions of hispolon on melanogenesis and apoptosis in B16-F10 melanoma cells. The results demonstrated that hispolon is not an enzymatic inhibitor for tyrosinase; rather, it represses the expression of tyrosinase and the microphthalmia-associated transcription factor (MITF) to reduce the production of melanin in α-melanocyte-stimulating hormone (α-MSH)-stimulated B16-F10 cells at lower concentrations (less than 2 μM). In contrast, at higher concentration (greater than 10 μM), hispolon can induce activity of caspase-3, -8 and -9 to trigger apoptosis of B16-F10 cells but not of Detroit 551 normal fibroblast cells. Therefore, we suggest that hispolon has the potential to treat hyperpigmentation diseases and melanoma skin cancer in the future.

## 1. Introduction

Melanin is produced by melanocytes for the protection of the skin against harmful ultraviolet (UV) irradiation; however, over-produced melanin frequently causes hyperpigmentation [1]. Melanogenesis is initiated with the first step of tyrosine oxidation by tyrosinase, which is a copper-containing and rate-limiting enzyme that augments melanin production by the hydroxylation of tyrosine into dihydroxyphenylalanine (DOPA) and also by the oxidation of DOPA into dopaquinone [2]. Furthermore, α-melanocyte-stimulating hormone (α-MSH) is required for the development of melanin in human skin through UV irradiation. The α-MSH binds to its specific receptor (melanocortin 1 receptor; MC1R) and increases cAMP, which activates a key transcription factor, microphthalmia-associated transcription factor (MITF). Tyrosinase and tyrosinase-related proteins (TRPs) are transcriptionally regulated by MITF [3,4]. Therefore, inhibition of tyrosinase activity and its expression is the most general approach to attain skin hypopigmentation, while the development of novel, potent and safe depigmentation agents is of great importance for medical, food and cosmetic products [5,6].

Melanoma arises from melanocytes, which are a very invasive type of tumor that migrate from vascular and lymphatic systems to form tumor tissue elsewhere in the body. Melanoma is one of the fastest-growing malignant tumor types [7,8]. Moreover, melanoma is often characterized by resistance to cytotoxic agents, which contributes to the high morbidity and mortality rates in patients [9]. For this reason, it is important to look for new anti-cancer agents that apply cytotoxicity activity against melanoma cells.

**Figure 1.**
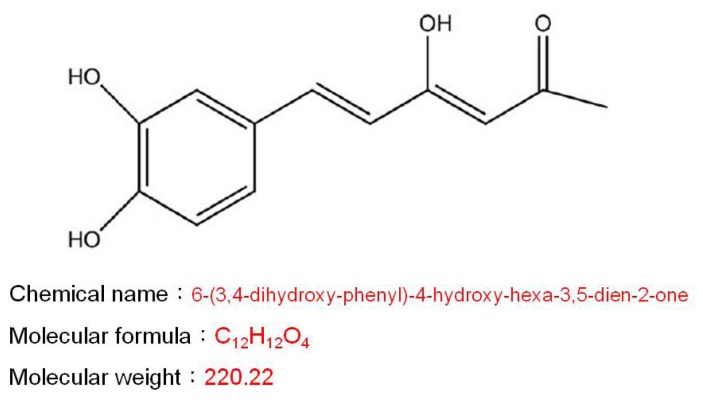
Chemical structure of hispolon.

*Phellinus linteus* (Berkeley & Curtis) Teng is a famous medical fungus of the genus *Phellinus* in the family of Hymenochaetaceae. It has been used as a traditional medicine in oriental countries for many years to treat various diseases, such as gastroenteric disorders, inflammation, tumors and lymphatic diseases [5,10]. Various bioactive components, such as polysaccharides, proteoglycans, furan derivatives, hispidin and hispolon, have been identified from *P. linteus* [11–13]. Among them, hispolon is one of the most important functional compounds (Figure 1).

Hispolon has antioxidant, anti-inflammatory and antiproliferative effects [9,14,15]. It protects against acute liver damage in rats by inhibiting lipid peroxidation, pro-inflammatory cytokine and oxidative stress [16]. Furthermore, several reports have demonstrated that hispolon exhibits anticancer effects through the inhibition of cell growth or metastasis in various types of tumor cells [17–19]. However, there is no study to date that discusses the effects of hispolon on hypopigmentation and anti-melanoma cells. Therefore, in the present study, we analyzed the functions of hispolon on the α-MSH-induced melanogenesis in B16-F10 melanoma cells to confirm the potential of hispolon as a depigmentation agent. We also analyzed the effect of hispolon on apoptosis in B16-F10 melanoma cells to verify the possibility of hispolon as an anti-melanoma agent. Furthermore, the proposed mechanisms by which hispolon functions were also discovered in this study.

## 2. Results and Discussion

### 2.1. Effects of Hispolon on Cell Viability

**Figure 2.**
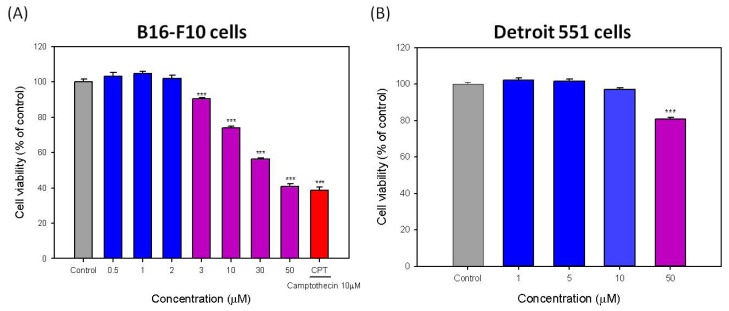
Effects of hispolon on the cell viability of (**A**) B16-F10 cells and (**B**) Detroit 551 cells. Each value is expressed as the mean ± S.E. (*n* = 3), *** *p* <0.001 compared with the control. CPT, camptothecin.

In the present study, we investigated the effect of hispolon on melanogenesis and apoptosis in B16-F10 melanoma cells. Accordingly, in the beginning, the cytotoxicities of hispolon on B16-F10 and Detroit 551 cells were evaluated. The results are shown in Figure 2. For B16-F10 cells, the cytotoxicity of hispolon was observed with a concentration of only 3 μM. Moreover, the cell viability of B16-F10 cells decreased to only approximately 40.8% (Figure 2A). The calculated IC_50_ value is 41.5 ± 1.3 μM. The positive control, camptothecin (CPT), reduced the cell viability of B16-F10 cells to 38.7% at 10 μM. In contrast, at a concentration of 50 μM of hispolon, the cell viability of Detroit 551 cells remained at 80.8% (Figure 2B). Consequently, these results demonstrated that hispolon has greater cytotoxicity to B16-F10 melanoma cells. Moreover, if its concentration is greater than 10 μM, the cell viability of melanoma cells can clearly decrease. The anticancer compound camptothecin (CPT) has been demonstrated to be effective against a broad spectrum of tumors. Its molecular target has been established to be human DNA topoisomerase I. However, CPT can cause a number of toxic side effects to normal tissues [20]. Although the cytotoxicity of hispolon is not greater than that of CPT at the same concentration (10 μM), we can still suppose that hispolon has obvious cytotoxicity to B16-F10 melanoma cells at a higher concentration.

### 2.2. Effects of Hispolon on the Melanin Content and Cellular Tyrosinase Activity in α-MSH-Stimulated B16-F10 Cells

**Figure 3.**
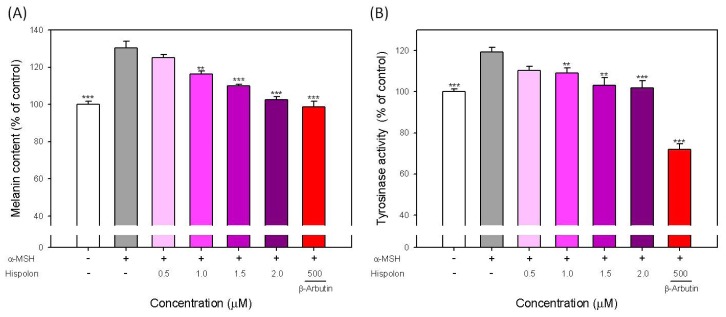
Effects of hispolon on the (**A**) melanin content and (**B**) cellular tyrosinase activity in α-melanocyte-stimulating hormone (α-MSH)-stimulated B16-F10 cells. Each value is expressed as the mean ± S.E. (*n* = 3). ** *p* < 0.01 and *** *p* < 0.001 compared with the α-MSH-stimulated group.

To evaluate the effect of hispolon on the melanogenesis of B16-F10 cells, we tested the function of hispolon on the melanin content and tyrosinase activity in α-MSH-stimulated B16-F10 cells. The results are shown in Figure 3. The level of melanin content in α-MSH-stimulated B16-F10 cells increased to approximately 130.4% of the untreated control group. Hispolon can evidently attenuate the α-MSH-stimulated melanin production in a dose-dependent manner (Figure 3A). When B16-F10 cells were treated with 2 μM of hispolon, the stimulated melanin content decreased to approximately 102.7% of the control. Moreover, 500 μM of arbutin also reduced the melanin content to 98.8% of the control (Figure 3A).

For tyrosinase activity, the stimulation of α-MSH increased the activity to approximately 120% of control. The treatments of hispolon also clearly decreased the stimulated tyrosinase activity in a dose-dependent manner (Figure 3B). However, the inhibition effect of arbutin on tyrosinase activity is more obvious than that on melanin content. The tyrosinase activity in B16-F10 cells treated with 500 μM of arbutin was reduced to only 72.1% of the control (Figure 3B). This difference might be because arbutin is a potent tyrosinase competitive inhibitor that directly inhibits the activity of tyrosinase. Thus, the decreasing proportion of melanin content is different to that of tyrosinase activity (Figure 3). However, from these results, we can still conclude that hispolon at lower concentrations has an effect on the inhibition of melanogenesis.

### 2.3. Direct Effects of Hispolon on the Activity of Mushroom Tyrosinase and Cellular Tyrosinase

**Figure 4.**
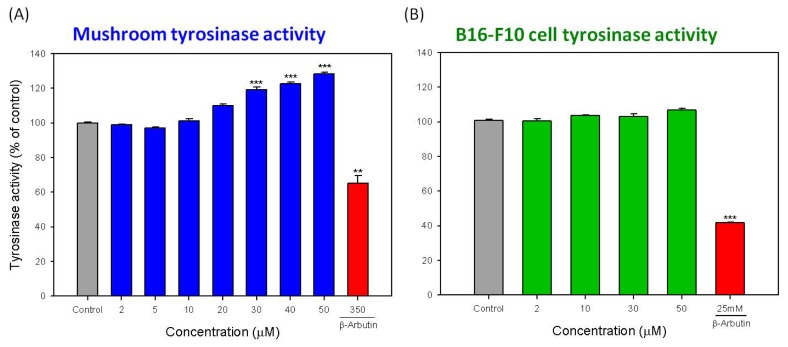
Direct effects of hispolon on the activity of (**A**) mushroom tyrosinase and (**B**) B16-F10 cellular tyrosinase. Each value is expressed as the mean ± S.E. (*n* = 3). ** *p* < 0.01 and *** *p* < 0.001 compared with the control.

To determine whether the inhibition effect of hispolon on melanogenesis is mediated by the direct inhibition of tyrosinase activity, we tested the effects of hispolon on purified mushroom tyrosinase and extracted B16-F10 cellular tyrosinase. The results are shown in Figure 4. The tested hispolon concentrations increased from 2–50 μM. However, the tyrosinase activity of both the mushroom and B16-F10 cells did not decrease. Moreover, for mushroom tyrosinase, the activity was enhanced with an increase in the hispolon concentration. The enhanced mushroom tyrosinase activity was improved to 128.4% of the control at 50 μM of hispolon (Figure 4A). Besides, the inhibition effects of β-arbutin on mushroom tyrosinase (350 μM) and B16-F10 cellular tyrosinase (25 mM) were obvious, and the inhibition effect decreased the cellular tyrosinase activity to only approximately 40% of the control (Figure 4). Although the activity of B16-F10 cellular tyrosinase was slightly enhanced with an increasing hispolon concentration, the effect of hispolon on both the mushroom and B16-F10 cellular tyrosinase activity was improved (Figure 4). Thus, these results demonstrated that the suppression effect of hispolon on melanogenesis is not mediated by the direct inhibition of tyrosinase activity. Furthermore, hispolon has a slight effect on the enhancement of tyrosinase activity.

### 2.4. Effects of Hispolon on the Protein Level of Tyrosinase and MITF in α-MSH-Stimulated B16-F10 Cells

**Figure 5.**
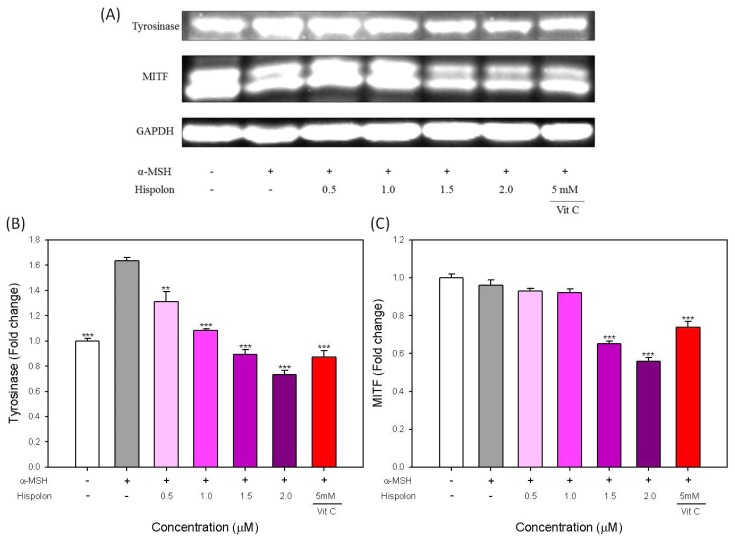
Effects of hispolon on the (**A**) protein level of tyrosinase and MITF in α-MSH-stimulated B16-F10 cells; The quantified results of (**B**) tyrosinase and (**C**) MITF. VitC, vitamin C. Each value is expressed as the mean ± S.E. (*n* = 3). ** *p* < 0.01 and *** *p* < 0.001 compared with the α-MSH-stimulated group.

Hispolon is not an enzymatic inhibitor for tyrosinase. Therefore, we analyzed the effects of hispolon on the protein level of tyrosinase and MITF in α-MSH-stimulated B16-F10 cells. The results of a western blot analysis are shown in Figure 5A. The quantified protein levels of tyrosinase and MITF are shown in Figure 5B,C, respectively. For tyrosinase, α-MSH-stimulated tyrosinase expression in B16-F10 cells is clearly attenuated by the addition of hispolon in a dose-dependent manner (Figure 5A,B). When the concentrations of hispolon were greater than 1.5 μM, the protein content of tyrosinase was reduced to lower levels than that of the untreated control group. Furthermore, vitamin C at a relatively high concentration (5 mM) can also clearly reduce the expression of tyrosinase (Figure 5A,B). A similar result for MITF is presented in Figure 5A,C. The protein levels of MITF in B16-F10 cells were reduced by the addition of hispolon. Although the reduction was only clearly observed at hispolon concentrations greater than 1.5 μM, the reduced MITF levels decreased to only 61.7%–58.9% of the control, which were less than that of the group treated with 5 mM of vitamin C (Figure 5C). Moreover, the protein level of MITF in B167-F10 cells was not stimulated by α-MSH in our experiment, possibly because the induction time of α-MSH treatment is 24 h; thus, the effect of α-MSH on MITF may have already been negated.

Many compounds, such as arbutin, deoxyarbutin, kojic acid, aloesin, carthamus yellow and 4-*n*-butylresorcinol, can suppress melanogenesis through direct inhibition of tyrosinase activity [21–24]. However, some compounds, such as acetylsalicylic acid (aspirin), kaempferide and vanillic acid, are not enzymatic inhibitors for tyrosinase and exert hypopigmentation by the downregulation of MITF and tyrosinase expression [25–27]. Also, hispolon is not an enzymatic inhibitor for tyrosinase. Our results demonstrated that hispolon at lower concentrations (less than 2 μM) also suppresses the expression of tyrosinase in inhibiting the production of melanin in α-MSH-stimulated B16-F10 cells. Moreover, the downregulation of tyrosinase expression is mediated by the suppression of its specific transcription factor, MITF.

### 2.5. Effects of Hispolon on Apoptosis in B16-F10 and Detroit 551 Cells

**Figure 6.**
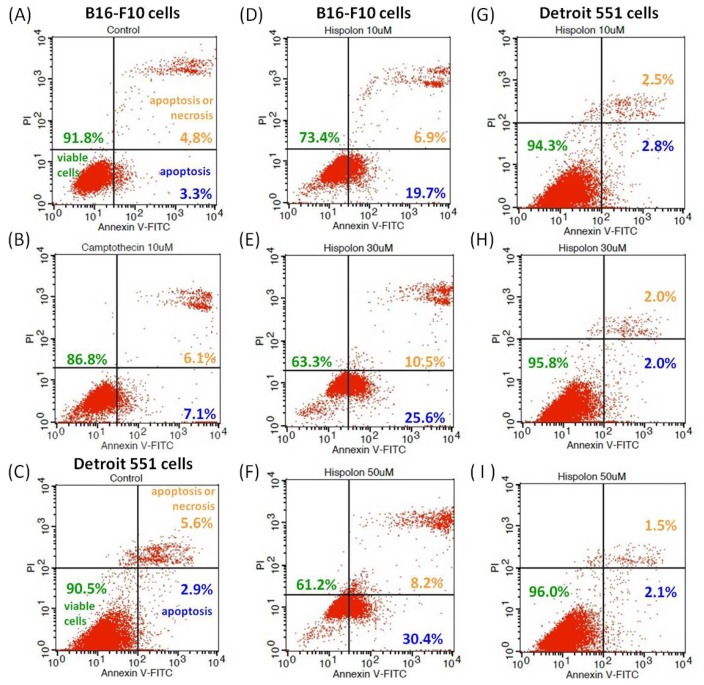
Effects of hispolon on apoptosis in B16-F10 and Detroit 551 cells. The cells were stained with fluorescein isothiocyanate (FITC)-labeled annexin-V/propidium iodide (PI) double stain, and the percentages of apoptotic and necrotic cells were calculated. (**A**) untreated B16-F10 cells; (**B**) 10 μM camptothecin treated B16-F10 cells; (**C**) untreated Detroit 551 cells; (**D**) B16-F10 cells treated with 10 μM of hispolon; (**E**) B16-F10 cells treated with 30 μM of hispolon; (**F**) B16-F10 cells treated with 50 μM of hispolon, (**G**) Detroit 551 cells treated with 10 μM of hispolon; (**H**) Detroit 551 cells treated with 30 μM of hispolon; and (**I**) Detroit 551 cells treated with 50 μM of hispolon.

At higher-concentration conditions, hispolon has obvious cytotoxicity to B16-F10 cells (malignant melanoma cells) but not Detroit 551 cells (normal fibroblast cells). Therefore, we further analyzed the effects of hispolon on apoptosis in B16-F10 and Detroit 551 cells. The cells were stained with fluorescein isothiocyanate (FITC)-labeled annexin-V/propidium iodide (PI) double stain, and the percentages of apoptotic and necrotic cells were calculated. The results are shown in Figure 6. For untreated B16-F10 (Figure 6A) and Detroit 551 cells (Figure 6B), more than 90% of the cells were viable and healthy in the culture, and only 2.9%–3.3% of the cells were apoptotic. However, hispolon-treated B16-F10 cells (Figure 6D–F) clearly had more apoptotic cells than the untreated group. As the concentration of hispolon was increased from 10–50 μM, the percentage of apoptotic cells also increased from 19.7%–30.4%. In contrast, hispolon-treated Detroit 551 cells had the almost the same ratio of apoptotic cells as the control group, in which the percentages of apoptotic cells were only 2.0%–2.8% (Figure 6G–I). Furthermore, the B16-F10 cells treated with 10 μM of camptothecin had a relatively lower fraction of viable cells, but this effect was stronger than that of hispolon (Figure 6B).

Many effective anticancer compounds can induce apoptosis of malignant tumor cells, such as camptothecin, cisplatin, gemcitabine and curcumin [28–30]. However, one of the most important issues for anticancer agents is safety. If a functional anticancer agent induces apoptosis in tumor cells but not in normal cells, it can be considered a relatively harmless anticancer agent. The anticancer effects of *P. linteus* extract have been demonstrated by the inhibition of invasive melanoma B16-BL6 cells [31]. Thus, in our results, it is not surprising that hispolon has a cytotoxicity effect on B16-F10 cells, which induces the apoptosis. Moreover, this induction of apoptosis is not observed in Detroit 551 cells.

### 2.6. Effects of Hispolon on the Activation of Caspase-3, -8 and -9 in B16-F10 Cells

**Figure 7.**
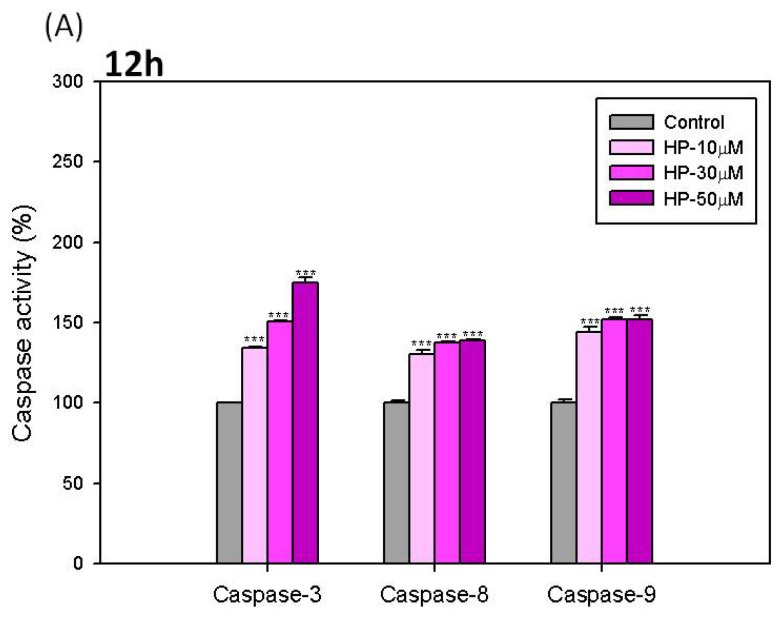

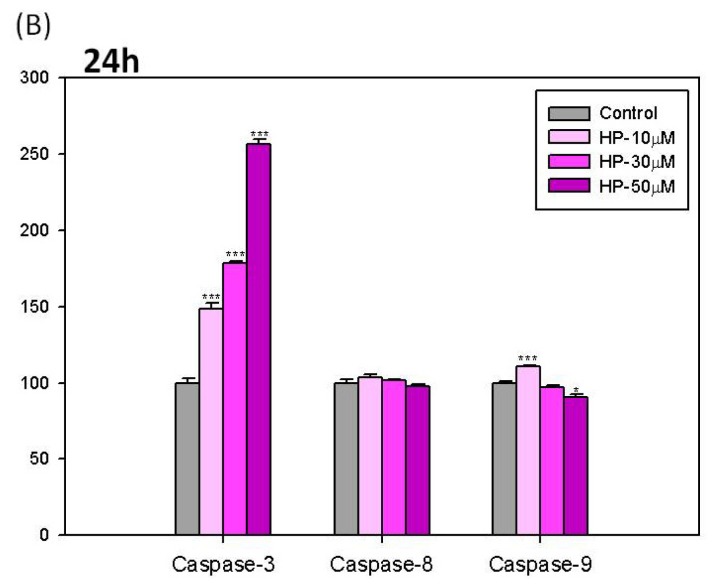
Effects of hispolon on the activation of caspase-3,-8 and -9 in B16-F10 cells at (**A**) 12 h and (**B**) 24 h. Each value is expressed as the mean ± S.E. (*n* = 3). * *p* < 0.05 and *** *p* < 0.001 compared with the control.

To further confirm the apoptosis-induction effect of hispolon on B16-F10 cells, we analyzed the activity of apoptotic enzymes, caspase-3, -8 and -9, in hispolon-treated B16-F10 cells. The results are shown in Figure 7. In the 12-h treatment of hispolon, the activities of caspase-3, -8 and -9 in B16-F10 cells all increased in a dose-dependent manner (Figure 7A). Furthermore, at 24 h, the activity of caspase-3 was enhanced by hispolon to the highest levels, approximately 150%–250% of the control. However, the activities of caspase-8 and -9 recovered to a normal level (Figure 7B).

Caspases are the executioners of apoptosis and play key roles in apoptotic cell death. Caspases have been divided into two groups based on their functions in apoptosis. One group is the initiator caspases, which include caspase-8 and -9, whereas the other group is the effector caspases, which include caspase-3 [32]. Furthermore, two classical signaling pathways of apoptosis, the extrinsic pathway and the intrinsic pathway, are well known. The extrinsic pathway is also called the death-receptor-dependent pathway. This pathway is activated by caspase-8. In contrast, the intrinsic pathway is also known as the mitochondria-dependent pathway, which is triggered by cellular damages or stress, and the activation caspase-9 is involved in its signal transduction [33,34]. In our results, hispolon induced the activation of both caspase-8 and -9 at 12 h, and these signals subsequently triggered the highest activity of caspase-3 at 24 h in B16-F10 cells. These results suggest that hispolon at higher concentrations (greater than 10 μM) can induce the activities of caspase-3, -8 and -9 to trigger the apoptosis of B16-F10 cells.

## 3. Experimental Section

### 3.1. Materials

Hispolon was purchased from Enzo Life Sciences, Inc. (Farmingdale, NY, USA). Camptothecin, vitamin C, mushroom tyrosinase, L-DOPA, α-MSH, dimethyl sulfoxide (DMSO), sodium hydroxide (NaOH), sodium dodecyl sulfate (SDS) and other chemicals were purchased from Sigma-Aldrich (St. Louis, MO, USA). Dulbecco’s modified Eagle’s medium (DMEM), α-modified essential medium (α-MEM), fetal bovine serum (FBS), penicillin, streptomycin and trypsin-EDTA were purchased from Gibco BRL/Invitrogen (Carlsbad, CA, USA). The 3-(4,5-dimethylthiazol-2-yl)-2,5-diphenyl tetrazolium bromide (MTT) was purchased from Affymetrix/USB (Cleveland, OH, USA). The β-arbutin was purchased from Alfa Aesar (Ward Hill, MA, USA). The anti-tyrosinase, anti-MITF and anti-glyceraldehyde-3-phosphate dehydrogenase (GAPDH) antibodies were purchased from Santa Cruz Biotechnology (Santa Cruz, CA, USA). The FITC-labeled annexin-V/PI apoptosis detection kit was purchased from BD Biosciences (San Jose, CA, USA). Caspases colorimetric assay kit was purchased from BioVision, Inc. (Milpitas, CA, USA). Deionized distilled water (ddH_2_O) for solutions and buffers was obtained from the Milli-Q system (Millipore, Bedford, MA, USA).

### 3.2. Cell Line and Cell Culture

The Detroit 551 normal fibroblast cells (BCRC 60118) and the B16-F10 melanoma cells (BCRC 60031) were purchased from the Food Industry Research and Development Institute (FIRDI, Hsinchu, Taiwan). The Detroit 551 and B16-F10 cells were cultured in α-MEM and DMEM, respectively, and then supplemented with 10% FBS, 2 mM glutamine, 100 mg/mL streptomycin and 100 U/mL penicillin. The cells were maintained in a humidified incubator with 5% CO_2_ at 37 °C, and they were sub-cultured every 3 to 4 days to maintain logarithmic growth.

### 3.3. MTT Assay for Cell Viability

Detroit 551 and B16-F10 cells were seeded in 96-well plates (6 × 10^3^ and 5 × 10^3^ cells/well) using an α-MEM or DMEM medium supplemented with 10% FBS for 24 h, after which the medium was replaced with an FBS-free medium for another 24 h. The prepared cells were subsequently treated with different concentrations of samples for 48 h. Next, 100 μL (0.5 mg/mL) of MTT solution was added to cells, which were then incubated at 37 °C for 30 min and washed twice with phosphate-buffered saline (PBS). Finally, the cleaned cells were lysed with 100 μL of DMSO, and the absorbance was measured spectrophotometrically at 540 nm using an ELISA reader. Camptothecin (10 μM) was used as the control. The inhibitory concentration value at 50% cell viability is defined as IC_50_.

### 3.4. Melanin Content Analysis

For melanin content analysis, B16-F10 cells (8 × 10^4^ cells/well) were incubated in 6-well plates with 50 nM α-MSH for 24 h. The cells were then treated with various concentrations of the test samples for 24 h. After this treatment, the cells were dissolved in 120 μL of 1 N NaOH for 1 h at 65 °C to solubilize the melanin. The total amount of melanin in each cell suspension was determined by recording the absorbance at 405 nm. The melanin content was calculated and corrected for the cell number.

### 3.5. Tyrosinase Activity Assays

For cellular tyrosinase activity assays, B16-F10 cells (3 × 10^5^ cells/well) were incubated in 10 cm plates with 50 nM α-MSH for 24 h. The cells were then treated with various concentrations of the test samples for 24 h. After this treatment, the cells were lysed at 4 °C for 20 min and the cellular proteins were isolated by centrifugation at 10,000× *g* for 3 min. The quantified (via the Bradford method [35]) cellular protein with 190 μL (800 μg of protein) was added to 10 μL of L-DOPA (10 mM) at 37 °C for 30 min. The spectrophotometric analysis was performed at 475 nm and the formation of DOPAchrome was calculated as the percentage of enzyme activity. β-arbutin (500 μM) was used as control.

For the direct tyrosinase activity assay, each 60-μL test sample was mixed with 10 μL of L-DOPA (10 mM). Then, 40 μL of mushroom tyrosinase solution (100 units/mL) or B16-F10 cellular tyrosinase (800 μg of protein) were added to the mixture, which was then incubated for 25 min at 37 °C. The spectrophotometric analysis was performed at 475 nm and the formation of DOPAchrome was calculated as the percentage of enzyme activity. β-arbutin (25 mM) was used as the control.

### 3.6. Western Blot Analysis

The B16-F10 cells (3 × 10^5^ cells/well) were incubated in 6-well plates with 50 nM of α-MSH for 24 h. The cells were then treated with various concentrations of the test samples for 6 h. After this treatment, the cells were sonicated with a 200-μL lysis buffer at 4 °C for 20 min, and the lysates were collected and then quantified. For western blotting, each well was loaded with 20 ng of protein and resolved by 10% SDS PAGE and then electrotransferred on to a polyvinylidene difluoride (PVDF) membrane using the Bio-Rad MiniProtean II apparatus (Bio-Rad Laboratories, Carlsbad, CA, USA). The blots were subsequently incubated with an anti-tyrosinase, anti-MITF or anti-GAPDH antibody as the primary antibody, and the immune complexes were visualized using an ECL reagent (Millipore, Billerica, MA, USA). The bands were scanned and then quantified by measuring the optical densities using the VIpro Platinum 1.1 software package (Version 12.9; UVItec, Cambridge, UK). Vitamin C (5 mM) was used as the control.

### 3.7. Annexin-V/PI Staining Assay

Apoptosis-mediated cell death was examined by a double-staining method with an FITC-labeled annexin-V/PI apoptosis detection kit. For PI and annexin-V double staining, B16-F10 cells (3 × 10^5^ cells/well) or Detroit 551 cells (4 × 10^5^ cells/well) were treated with samples for 24 h. The cells were then collected and stained with FITC-labeled annexin-V and PI according to the manufacturer’s protocols (BD Biosciences). Apoptotic cells were analyzed with a flow cytometer, the BD FACScan flow cytometric system (BD Biosciences, San Jose, CA, USA). Data acquisition and analysis were performed by the CellQuest software package (BD Biosciences). Camptothecin (10 μM) was used as the control.

### 3.8. Caspases Activity Assays

Cellular caspase activity was measured by the chromophore-labeled specific substrates, including Asp-Glu-Val-Asp p-nitroanilide (DEVD-pNA, the substrate for caspase-3), Ile-Glu-Thr-Asp p-nitroanilide (IETD-pNA, the substrate for caspase-8) and Leu-Glu-His-Asp p-nitroanilide (LEHD-pNA, the substrate for caspase-9). B16-F10 cells (3 × 10^5^ cells/well) were incubated in 10-cm plates with 50 nM of α-MSH for 24 h. The cells were then treated with samples for 12 or 24 h. After the treatments, the cells were sonicated with a 200-μL lysis buffer at 4 °C for 20 min. The cellular protein was then quantified and analyzed with the chromophore-labeled specific substrates according to the manufacturer’s protocols (BioVision, Inc., Milpitas, CA, USA). The spectrophotometric analysis was performed at 405 nm and the caspase activity was expressed as a percentage of the untreated control.

### 3.9. Statistical Analysis

Quantitative data were analyzed using Student’s *t*-tests and are presented as the mean ± standard error (S.E.) of three independent experiments. *p*-values less than 0.05 were considered to be significant.

## 4. Conclusions

**Figure 8.**
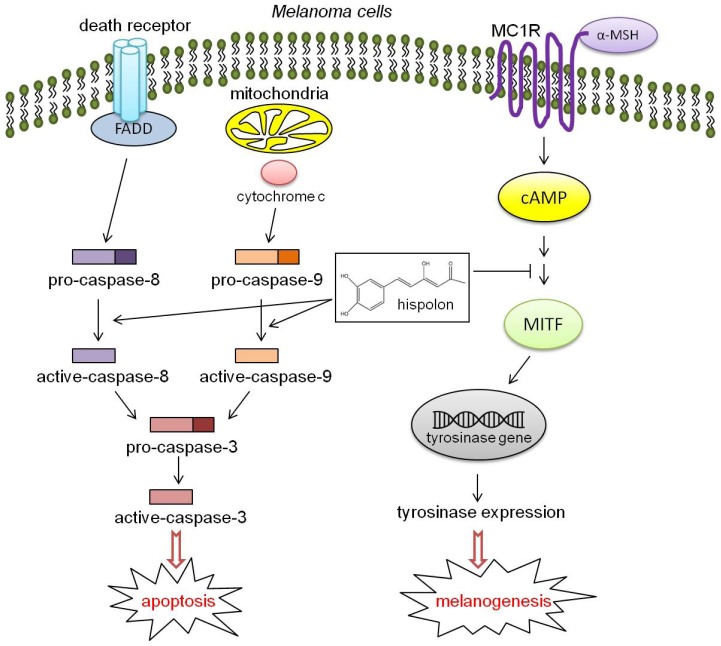
Proposed mechanisms for the effect of hispolon on melanogenesis and apoptosis in B16-F10 melanoma cells.

In summary, the proposed mechanisms of hispolon on melanogenesis and apoptosis in B16-F10 melanoma cells are shown in Figure 8. Hispolon is not an enzymatic inhibitor for tyrosinase; at lower concentrations (less than 2 μM), it represses the expression of tyrosinase and MITF to reduce the production of melanin in α-MSH-stimulated B16-F10 cells. At higher concentrations (greater than 10 μM), hispolon can induce the activity of caspase-3, -8 and -9 to trigger the apoptosis of B16-F10 cells but not Detroit 551 cells (Figure 8). Therefore, hispolon has the potential to be used as a hypopigmentation and anticancer agent in the future.
